# Gait variability as a dual-pathway marker of cognitive-motor dysfunction in older adults with type 2 diabetes mellitus

**DOI:** 10.3389/fendo.2026.1814718

**Published:** 2026-03-31

**Authors:** Zheping Zhou, Guiyu Li, Jiahao Chen, Zhaozhe Wang, Honghong Zhang, Yueju Wang, Ji Hu

**Affiliations:** 1 Department of Endocrinology and Metabolic Medicine, the Second Affiliated Hospital of Soochow University, Suzhou, China; 2 Department of Gerontology, the First Affiliated Hospital of Soochow University, Suzhou, China; 3 Department of Gerontology, Affiliated Changshu Hospital of Nantong University, Changshu, China

**Keywords:** cognitive function, diabetes, gait variability, mediation analysis, wearable sensors

## Abstract

**Background:**

Diabetes-related gait disorders are important drivers of falls and functional decline in older adults. Gait variability, as an indicator highly sensitive to fluctuations between steps, remains underexplored in diabetic populations. Compared with the average gait parameters, gait variability may better reflect impaired neuromuscular control and the risk of falling. This study aimed to evaluate the diagnostic accuracy of gait variability parameters, and test whether cognitive function plays an intermediary role between type 2 diabetes mellitus (T2DM) and gait variability.

**Methods:**

A total of 56 non-diabetic older participants and 37 T2DM patients (aged 60 years or older) were enrolled in this study. We used wearable inertial sensors to evaluate the gait parameters (including the variability in stance time, gait speed, stride length and turn duration) during straight walking and turning tasks. Standardized tools were used to evaluate cognitive functions, including the Mini-Mental State Examination (MMSE) and Montreal Cognitive Assessment (MoCA) and other verified measures.

**Results:**

Our results showed that T2DM patients exhibited significantly higher gait variability across all indicators. All gait variability indices were significantly negatively correlated with cognitive function (r = −0.20 to −0.53, P < 0.05). After adjusting for demographic characteristics and cognitive functions, the T2DM status was still an independent predictor of gait variability. The receiver operating characteristic (ROC) curve analysis showed that the stance time variability had good diagnostic accuracy (area under the curve [AUC] = 0.813, 95% CI 0.727–0.898, p < 0.001, sensitivity 94.6%), and gait speed variability also demonstrated good diagnostic performance (AUC = 0.801, 95% CI 0.705–0.897, p < 0.001). Mediation analysis showed that cognitive function mediated the effect of T2DM on stance time variability, and the mediated effect accounted for 31.9% of the total effect.

**Conclusion:**

This study showed that T2DM patients demonstrated a significant increase in gait variability. This variation was closely associated with cognitive decline. Stance time and gait speed variability could be used as a sensitive and non-invasive screening method to identify gait dysfunction related to diabetes. T2DM may affect gait stability through dual pathways, involving both cognitive decline and non-cognitive mechanisms. Comprehensive intervention strategies (including blood sugar control, neuropathy management and cognitive training) could improve the gait stability of T2DM elderly people and mitigate the risk of falling.

## 1 Introduction

The global burden of diabetes has posed a substantial public health challenge. About 589 million adults around the world were affected in 2024, and the number is expected to increase to 853 million by 2050 (1). In China, the prevalence of type 2 diabetes mellitus (T2DM) in people aged 60 and above is more than 30%; in this particular population, beyond its fully recognized metabolic complications (including cardiovascular diseases, kidney disease and retinopathy), T2DM is also increasingly considered to be a significant contributor to functional decline and disability in aging populations (2). Among the various manifestations of diabetes-related functional impairment, gait disorders have received growing attention because of its negative impact on mobility, fall risk and overall quality of life (3). T2DM individuals often exhibit altered gait characteristics, such as reduced gait speed, shortened stride length and fewer acceleration patterns. Compared with non-diabetic peers, these changes collectively increase the risk of falling (4). These gait abnormalities are thought to be caused by a variety of pathophysiological mechanisms, including diabetic peripheral neuropathy (DPN) - sensory feedback that is crucial to gait stability (5), and diabetes-related muscle weakness, lower limb muscle strength decline and proprioception impairment (6). In addition, emerging evidence suggests that the central nervous system complications of T2DM may also further aggravate gait dysfunction by disrupting the cortical network involved in motor control and executive functions (7). Existing studies on T2DM-related gait impairment mostly focus on the average parameters of gait, but ignore the clinical value of gait variability.

Traditional gait evaluation mainly relies on mean spatiotemporal parameters such as gait speed and stride length. Accumulating evidence shows that gait variability (quantified by the coefficient of variation, CV, or standard deviation, SD, of stride-to-stride fluctuations) may be a more sensitive and clinically relevant indicator reflecting the neurological integrity and the risk of falling (8, 9). Gait variability is considered to reflect the complex interaction between sensory input, central processing and motor output. Increased gait variability suggests that neuromuscular control is impaired, and the adaptability to environmental demands is reduced (10, 11). In populations with neurological diseases such as Parkinson’s disease and Alzheimer’s disease, increased gait variability has been proven to be more accurate in predicting falls, cognitive decline and death than average gait parameters (12, 13). Although the prevalence of gait disturbances and cognitive impairment in T2DM populations is relatively high (14), the relationship between T2DM and gait variability is still insufficiently studied. Most research focuses on the basic gait parameters of straight-line walking tasks, and pays limited attention to the parameters in complex walking conditions that require greater postural control and executive functions (15). Previous studies have shown that T2DM is associated with accelerated cognitive aging and an increased risk of dementia (16). However, the role of cognitive function as a potential mediating factor connecting T2DM and gait variability has not been systematically investigated. Because gait variability in neurodegenerative diseases reflects cognitive and cortical deterioration, cognitive decline may represent a critical mechanistic pathway linking T2DM to impaired gait stability (13, 17). To effectively prevent falls and maintain mobility in the rapidly growing T2DM elderly population, it is imperative to transform the above understanding into targeted screening and intervention protocols.

This study employed wearable gait analysis devices and comprehensive cognitive assessment to explore the gait variability of older adults with T2DM and its relationship with cognitive function. We characterized the gait parameters and gait variability in T2DM elderly during straight-line walking and turning. Our research analyzed the correlation between gait variability and cognitive performance, and evaluated the diagnostic accuracy of gait variability parameters. The mediating role of cognitive function between T2DM and gait variability was tested through mediation analysis, so as to explain the potential mechanism of gait stability impairment via the dual pathways involving peripheral neuropathy and central cognitive decline. Based on these objectives, we hypothesized that older adults with T2DM would exhibit greater gait variability than controls, that poorer cognitive performance would be associated with greater gait variability, and that cognitive function would partially mediate the association between T2DM and gait variability.

This study may provide objective and quantifiable indicators for the early clinical identification of older adults with T2DM gait instability and high risk of falling. These findings may provide a theoretical basis for the integration of gait variability into routine functional evaluation and stratified management. At the same time, these findings may help inform the development of a comprehensive intervention strategy that combines peripheral neuropathy management and cognitive training in order to improve gait stability and reduce the risk of adverse events.

## 2 Methods

### 2.1 Study design and participants

Participants aged 60 years or older were consecutively recruited from the outpatient Geriatrics Clinic at the First Affiliated Hospital of Soochow University. Eligible individuals could walk independently for 10 minutes without assistance and had no severe systemic comorbidities. This study was conducted in accordance with the principles of the Declaration of Helsinki and was approved by the Ethics Committees of the First and Second Affiliated Hospitals of Soochow University. Informed consent was obtained from all individual participants included in the study.

T2DM was determined by an endocrinologist based on outpatient records in accordance with American Diabetes Association (ADA) criteria (18). A second reviewer verified diagnoses; For participants with T2DM, additional inclusion criteria included (1): physician-diagnosed T2DM according to ADA criteria (2); absence of acute hyperglycemic episodes (e.g., diabetic ketoacidosis, hyperosmolar hyperglycemic state) or severe hypoglycemia requiring medical intervention within the past 3 months (3); stable diabetes management with no changes in oral hypoglycemic agents or insulin regimen within 3 months prior to assessment.

Exclusion criteria included:(1)Neuromuscular disorders affecting gait (e.g., stroke, Parkinson’s disease) (2); Significant musculoskeletal disorders of the lower limbs (e.g., advanced arthritis, or a history of major trauma or orthopedic surgery) (3); Inability to cooperate with assessments due to mental health conditions or cognitive/behavioral symptoms, irrespective of diagnostic labels (4); Severe visual impairment (including moderate to severe non-proliferative or proliferative diabetic retinopathy documented by ophthalmologic examination) and hearing impairment (5); Cardiovascular, peripheral vascular, or respiratory disorders with functional limitation to ambulation (symptomatic heart failure, unstable angina, significant arrhythmias, chronic respiratory insufficiency, or severe peripheral arterial disease) (6); Recent use of medications with known effects on gait or cognition within 14 days prior to assessment, including but not limited to benzodiazepines, antipsychotics, tricyclics or other strongly anticholinergic agents, opioid analgesics, skeletal muscle relaxants, and sedative-hypnotics (7); Severe diabetic complications that could independently impair gait performance, including active diabetic foot ulcer, or Charcot neuroarthropathy (8); Other conditions likely to acutely alter gait stability or speed: acute illness (e.g., active infection, acute vertigo), severe pain flare, orthostatic hypotension with symptomatic instability, unplanned hospitalization or major treatment change within the preceding 2 weeks, or a documented fall within the preceding 3 months that resulted in persistent gait alteration.

Participants then completed a series of formal assessments, including a structured medical history interview, comprehensive neuropsychological testing and a functional assessment of activities of daily living. All clinical assessments and gait measurements were completed on the same day.

### 2.2 Sociodemographic characteristics and cognitive assessment

Sociodemographic data (age, sex, body measurements, education, medical history) was collected through direct interviews. Global cognition was assessed using both the Mini-Mental State Examination (MMSE) (19) and the Montreal Cognitive Assessment (MoCA; Beijing version), the latter incorporating a standard one-point correction for lower education (<12 years) (20). For the mediation analysis, MMSE was used to represent global cognitive function because it is widely used internationally and allows better cross-study comparison. In contrast, the Beijing version of MoCA includes adaptations specific to the Chinese population. Then, we evaluated specific cognitive domains: visuospatial/executive function with the Clock Drawing Test (21); episodic memory with the Auditory Verbal Learning Test (AVLT)-Chinese Version (22); and semantic fluency with a category naming task (animals) (23). Additionally, we used the Instrumental Activities of Daily Living (IADL) scale to measure functional independence (24). Grip strength was assessed as the average of two measurements of the dominant hand.

### 2.3 Gait assessment

**Figure 1:**
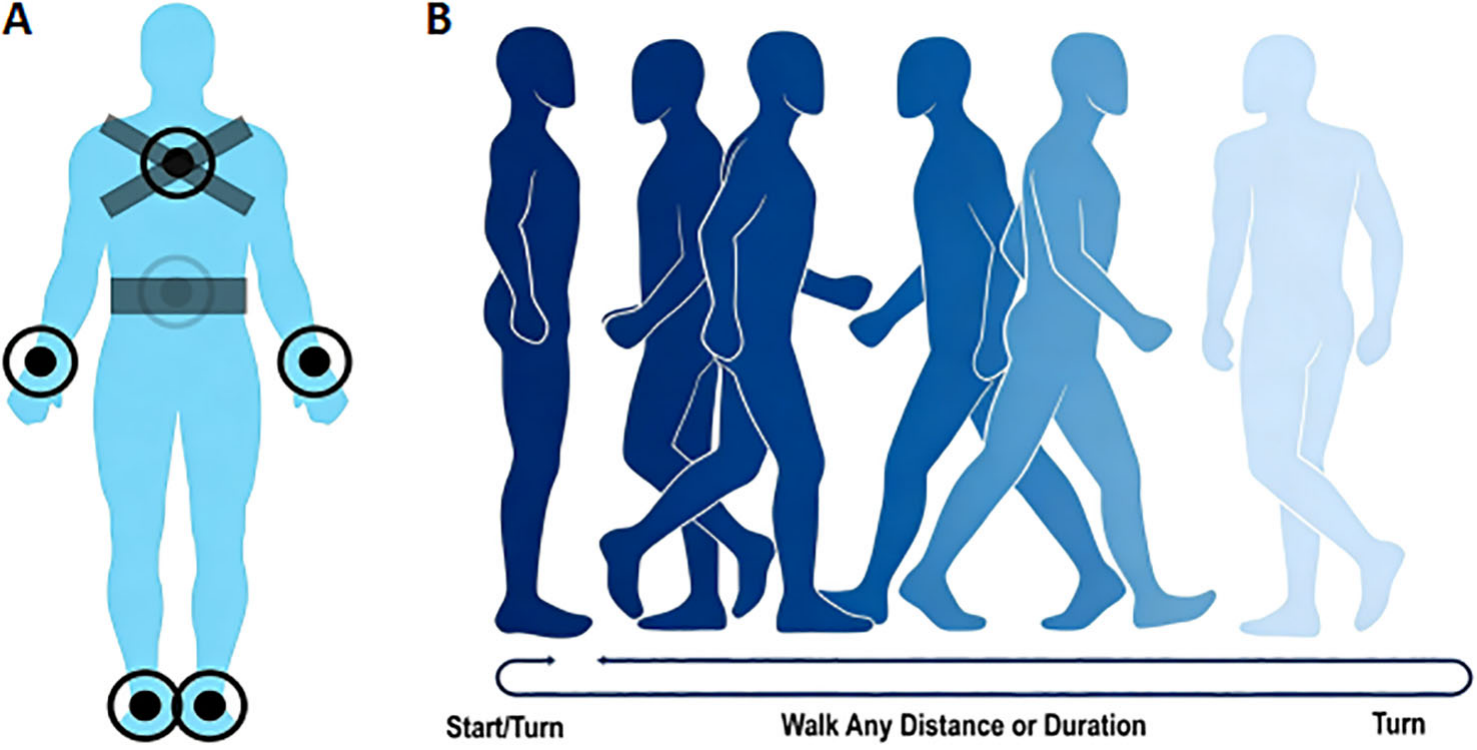
Wearable sensors placement on participants and protocols of the gait test. **(A)** Placement of six Opal sensors (low back, sternum, bilateral wrists, bilateral shanks). **(B)** Gait test protocol schematic (start/turn, walking phase, and turn).

Spatial and temporal gait parameters were quantified using a wireless inertial sensor system (APDM Inc., Portland, OR, United States). The system included triaxial accelerometers, gyroscopes, magnetometers, and a barometric pressure sensor. According to the manufacturer’s specifications, the sensors provided 14-bit resolution, a bandwidth of 50 Hz, an internal sampling rate of up to 1280 Hz, and an output rate of 20–128 Hz; the accelerometer range was ±6 g, the gyroscope range was ±2000°/s, and the magnetometer range was ±6 Gauss. The raw data were processed using APDM’s Mobility Lab™ (APDM, Inc) (25) to extract the gait features of interest. Six Opals were positioned on the participant’s body at the following locations: the low back, the sternum, bilateral wrists, and bilateral shanks (**Figure 1A**) and secured using the manufacturer’s elastic straps. To ensure consistency, sensor placement was performed by the same trained assessor according to standardized anatomical landmarks and checked before the recorded trial. Participants were instructed to walk at their self-selected usual pace with comfortable shoes on a 7-meter-long straight walkway (with start/end points marked by colored tape), execute a 180° turn, and return to the start (**Figure 1B**). Before the timed trial, all participants received standardized verbal instructions and a live demonstration of the walking course. Participants then performed one practice out-and-back walk to familiarize themselves with the procedure; data from the practice walk were not included in the analysis. The duration of each walking trial was set at 2 minutes. They were explicitly instructed to walk at their usual pace. With an experienced researcher walking behind for safety, participants then completed one recorded walking trial. We focused on gait parameters of interest for detailed analysis, and the corresponding gait variability values were calculated for 5 key gait parameters (stance, gait speed, stride length, turn duration, and turn velocity) using the formula: gait variability (%) = (standard deviation of gait parameter/mean of gait parameter) × 100%. For these five parameters, the mean and standard deviation were automatically generated by the software from the recorded gait cycles. Stance was defined as the percentage of the gait cycle during which the foot was on the ground; gait speed as the forward distance travelled during the gait cycle divided by gait cycle duration; stride length as the forward distance travelled by a foot during a gait cycle; turn duration as the duration of the turn; and turn velocity as the peak angular velocity during turning. Gait features were extracted automatically by the software over the full 2-minute walking task according to the system’s built-in algorithms. Ultimately, a total of 93 participants completed all assessments and were included in the final analysis.

### 2.4 Statistical analysis

To detect a large effect (d = 0.80) with 80% power (α= 0.05, two-tailed), the *a priori* calculated sample size was 52 participants, with 26 allocated to each group. Our sample of 93 participants exceeded this requirement. All reported p-values were two-sided, and p < 0.05 was considered statistically significant. All statistical analyses were performed using R software (version 4.5.1). Model assumptions were verified through diagnostic plots, including residual normality (Q-Q plots) and homoscedasticity (residual vs. fitted plots). Normally distributed variables were described as mean ± SD and compared between the control (CON) and T2DM groups using independent-samples t-tests. Non-normally distributed variables were described as median (interquartile range, IQR) and compared using Mann–Whitney U tests. We calculated Cohen’s d to quantify effect sizes for gait variability. According to standard conventions, these were interpreted as follows: 0.2 (small), 0.5 (medium), and 0.8 (large). The relationships between gait variability measures and cognitive function scores were examined using Pearson correlation coefficients. Correlation strength was interpreted as weak (|r| < 0.3), moderate (0.3 ≤ |r| < 0.7), or strong (|r| ≥ 0.7). Correlation matrices were visualized using heatmaps.

To examine the independent effect of diabetes status on gait variability measures while controlling for potential confounding factors, we conducted hierarchical multiple linear regression analyses. Three progressive models were constructed for each gait variability outcome. Multicollinearity was assessed using variance inflation factors (VIF < 5). Regression coefficients (β) with 95% confidence intervals (CI) were calculated to quantify the magnitude and direction of associations. Model fit was assessed using R² and adjusted R² values. These regression analyses were used to evaluate independent associations and were separate from the formal mediation analysis.

The discriminatory power of gait variability measures between CON and T2DM groups was assessed using the receiver operating characteristic (ROC) curve analysis. The area under the curve (AUC) with 95% CI was calculated for each measure using the DeLong method. The optimal cut-off values were determined using Youden’s index (sensitivity + specificity - 1), and the corresponding sensitivity and specificity were reported.

To examine whether cognitive function mediates the relationship between T2DM status and gait variability, we conducted regression-based causal mediation analyses using the mediation package in R. In the primary mediation model, T2DM status was specified as the independent variable, stance variability as the dependent variable, and MMSE score as the mediator. Age, sex, body mass index (BMI), and education were included as covariates in all mediation models. Specifically, a mediator model was fitted with MMSE as the outcome and T2DM as the predictor; an outcome model was fitted with stance variability as the outcome and both T2DM and MMSE as predictors; and a total effect model was fitted to estimate the overall association between T2DM and stance variability without inclusion of the mediator. The same set of covariates was consistently adjusted for across the mediator, outcome, and total effect models. The indirect effect (ACME), direct effect (ADE), total effect, and proportion mediated were estimated using nonparametric bootstrap procedures with 5,000 resamples. Statistical significance was assessed using percentile bootstrap 95% confidence intervals. An additional model without adjustment for education was performed as a supplementary sensitivity analysis.

## 3 Results

### 3.1 Participant characteristics

The baseline characteristics of the study participants are summarized in **Table 1**. A total of 93 participants were included in the analysis, comprising 56 CON individuals and 37 individuals with T2DM.

**Table 1:** Participant characteristics.

Category	Variable	CON (n=56)	T2DM (n=37)	p-value
Sample Size	N	56	37	—
Demographics	Age (years)	73.48 ± 7.47	74.54 ± 7.97	0.523
	Male, n (%)	33 (58.9%)	17 (45.9%)	0.218
	Education (years)	11.00 (8.00, 14.00)	8.00 (5.00, 11.00)	0.033*
	BMI (kg/m²)	22.82 ± 3.43	22.74 ± 3.06	0.897
	Hypertension, n (%)	20 (35.7%)	20 (54.1%)	0.078
Cognitive Function	MMSE (points)	27.00 (25.00, 28.00)	23.00 (14.00, 27.00)	< 0.001***
	MoCA (points)	24.00 (20.50, 26.50)	19.00 (11.00, 26.00)	< 0.001***
	Clock Drawing Test (points)	4.00 (3.00, 4.00)	3.00 (1.00, 4.00)	0.002**
	1-min Animal Fluency Test (points)	14.00 (12.00, 16.00)	13.00 (9.00, 15.00)	0.014*
	Instant Recall (points)	15.00 (12.00, 18.00)	9.00 (5.00, 14.00)	< 0.001***
	Short-term Recall (points)	4.00 (2.00, 6.00)	2.00 (0.00, 4.00)	< 0.001***
	Long-term Recall (points)	4.00 (2.00, 5.50)	0.00 (0.00, 4.00)	< 0.001***
Physical Functional	IADL (points)	12.00 (12.00, 13.00)	13.00 (12.00, 17.00)	0.011*
	Grip Strength (kg)	24.65 (20.20, 29.50)	18.10 (12.30, 27.10)	0.012*
Basic Gait Parameters	Gait Speed (cm/s)	78.49 ± 16.44	51.68 ± 15.97	< 0.001***
	Cadence (steps/min)	103.18 ± 9.82	87.07 ± 13.46	< 0.001***
	Stride Length (cm)	91.33 ± 18.03	71.66 ± 18.08	< 0.001***
	Gait Cycle Duration (s)	1.18 ± 0.12	1.44 ± 0.27	< 0.001***
	Lateral Step Variability (cm)	7.12 ± 3.00	6.25 ± 2.22	0.110
	Stance (%)	62.02 ± 2.05	64.53 ± 2.66	< 0.001***
	Swing (%)	37.98 ± 2.05	35.50 ± 2.67	< 0.001***
Turn Parameters	Turn Angle (degrees)	170.96 ± 14.32	148.94 ± 23.91	< 0.001***
	Steps in Turn (n)	4.11 ± 0.68	4.88 ± 4.54	0.317
	Turn Duration (s)	2.49 ± 0.32	2.61 ± 0.46	0.196
	Turn Velocity (°/s)	156.04 ± 35.29	121.06 ± 29.44	< 0.001***
Gait Variability	Gait Speed Variability (%)	9.61 ± 4.74	16.29 ± 7.48	< 0.001***
	Stride Length Variability (%)	7.04 ± 3.91	11.57 ± 5.42	< 0.001***
	Stance Variability (%)	2.19 ± 0.96	3.81 ± 1.75	< 0.001***
	Turn Duration Variability (%)	19.38 ± 6.76	26.87 ± 9.45	< 0.001***
	Turn Velocity Variability (%)	18.39 ± 15.10	22.00 ± 15.03	0.262

CON, control group; T2DM, type 2 diabetes mellitus; BMI, body mass index; MMSE, Mini-Mental State Examination; MoCA, Montreal Cognitive Assessment; IADL, Instrumental Activities of Daily Living. Continuous variables are presented as mean ± standard deviation (SD) or median (interquartile range, IQR); categorical variables are presented as n (%). Two-sided p-values were obtained using independent-samples t-tests for normally distributed continuous variables, Mann–Whitney U tests for non-normally distributed continuous variables, and chi-square tests or Fisher’s exact tests, as appropriate, for categorical variables. The bold values indicate p<0.05. *p < 0.05; **p < 0.01; **p < 0.001.

#### 3.1.1 Demographic characteristics

There was no significant age difference between the CON and T2DM groups (73.48 ± 7.47 years vs. 74.54 ± 7.97 years, P = 0.523). Gender distribution was also similar between groups (male: CON 58.9% vs. T2DM 45.9%, P = 0.218). BMI did not differ significantly between the two groups (CON: 22.82 ± 3.43 kg/m² vs. T2DM: 22.74 ± 3.06 kg/m², P = 0.897). However, the T2DM group had significantly fewer years of education compared to the CON group (median: 11.00 vs. 8.00 years; IQR: 8.00-14.00 vs. 5.00-11.00, P = 0.033).

#### 3.1.2 Cognitive function

Individuals with T2DM exhibited significantly poorer cognitive performance across multiple domains compared to CON. The T2DM group scored lower on MMSE (median: 27.00 vs. 23.00; IQR: 25.00-28.00 vs. 14.00-27.00, P < 0.001) and MoCA (median: 24.00 vs. 19.00; IQR: 20.50-26.50 vs. 11.00-26.00, P < 0.001).

Executive function measures revealed significant decline in the T2DM group, with lower scores on the Clock Drawing Test (median: 4.00 vs. 3.00; IQR: 3.00-4.00 vs. 1.00-4.00, P = 0.002) and the 1-minute Animal Fluency test (median: 14.00 vs. 13.00; IQR: 12.00-16.00 vs. 9.00-15.00, P = 0.014). Memory assessments demonstrated that the T2DM group performed significantly worse on Instant Recall (median: 15.00 vs. 9.00; IQR: 12.00-18.00 vs. 5.00-14.00, P < 0.001), Short-term Recall (median: 4.00 vs. 2.00; IQR: 2.00-6.00 vs. 0.00-4.00, P < 0.001), and Long-term Recall (median: 4.00 vs. 0.00; IQR: 2.00-5.50 vs. 0.00-4.00, P < 0.001).

#### 3.1.3 Physical function

IADL scores were significantly elevated in the T2DM group (median: 12.00 vs. 13.00; IQR: 12.00-13.00 vs. 12.00-17.00, P = 0.011). Grip strength was significantly lower in the T2DM group compared to the CON group (median: 24.65 vs. 18.10; IQR: 20.20-29.50 vs. 12.30-27.10, P = 0.012).

#### 3.1.4 Gait parameters and gait variability

T2DM patients exhibited a wide range of gait abnormalities, including abnormalities in gait parameters during straight and turning walking. Especially, the T2DM group demonstrated significantly higher gait variability across multiple parameters compared to the CON group. Gait Speed Variability was markedly elevated in the T2DM group (16.29 ± 7.48% vs. 9.61 ± 4.74%, P < 0.001), representing a 69% increase. Stride Length Variability was elevated in the T2DM group (11.57 ± 5.42% vs. 7.04 ± 3.91%, P < 0.001). Similarly, Stance Variability was also significantly higher in individuals with T2DM (3.81 ± 1.75% vs. 2.19 ± 0.96%, P < 0.001).

Turning-related gait variability measures showed similar patterns. Turn Duration Variability was significantly higher in the T2DM group (26.87 ± 9.45% vs. 19.38 ± 6.76%, P < 0.001), while Turn Velocity Variability did not differ significantly between groups (T2DM: 22.00 ± 15.03% vs. CON: 18.39 ± 15.10%, P = 0.262), though a trend toward increased variability was observed in the T2DM group.

Compared to CON, individuals with T2DM exhibited significantly higher variability across all spatiotemporal parameters. Gait variability measures differed significantly between groups (**Supplementary Figure S1**), with large effect sizes observed for most parameters (**Supplementary Figure S2**). Large effect sizes were observed for stance variability (d = 1.22), gait speed variability (d = 1.12), stride length variability (d = 0.99), and turn duration variability (d = 0.94), indicating substantial differences between groups. In contrast, turn velocity variability exhibited a negligible effect size (d = 0.24), consistent with the lack of statistical significance.

### 3.2 Correlation between gait variability and cognitive function

Correlation analyses revealed significant negative associations between all gait variability measures and cognitive performance scores (**Supplementary Figure S3**). Stance variability exhibited the strongest correlations, showing moderate-to-large negative relationships with MMSE (r = –0.50, P < 0.001), MoCA (r = –0.50, P < 0.001), and Instant Recall (r = –0.53, P < 0.001). Gait speed variability demonstrated a similar pattern, with correlations ranging from r = –0.31 (Clock Drawing, P < 0.01) to r = –0.50 (Instant Recall, P < 0.001). Stride length variability was moderately associated with all cognitive domains (r = –0.27 to –0.42, P < 0.01). Turn duration variability and turn velocity variability showed weaker but still significant associations across most measures (r = –0.20 to –0.43, P < 0.05 to P < 0.001).

Among cognitive tests, Instant Recall displayed the strongest correlations with gait variability (r = –0.27 to –0.53), followed by MMSE and MoCA (r = –0.26 to –0.50). Clock Drawing and Animal Fluency yielded smaller but significant coefficients for most gait parameters. These correlation results were descriptive and were not interpreted as formal mediation path coefficients.

### 3.3 Multiple linear regression analysis

Hierarchical multiple linear regression analyses revealed that T2DM status remained a significant independent predictor of multiple gait variability measures after adjusting for demographic and cognitive covariates (**Table 2**).

**Table 2:** Multiple linear regression analysis: effect of T2DM on gait variability.

Outcome variable	Model	Beta (β)	SE	95% CI	*t*	*P*-value	*R²*	Adj. *R²*
Gait Speed Variability	Model 1 (Unadjusted)	6.68	1.27	4.17–9.19	5.28	< 0.001	0.234	0.226
	Model 2 (Demographics)	7.03	1.29	4.47–9.60	5.45	< 0.001	0.278	0.245
	Model 3 (Full)	5.40	1.36	2.69–8.10	3.97	< 0.001	0.342	0.304
Stance Variability	Model 1 (Unadjusted)	1.62	0.28	1.06–2.18	5.75	< 0.001	0.266	0.258
	Model 2 (Demographics)	1.64	0.29	1.06–2.21	5.65	< 0.001	0.296	0.264
	Model 3 (Full)	1.12	0.29	0.54–1.70	3.85	< 0.001	0.420	0.387
Stride Length Variability	Model 1 (Unadjusted)	4.53	0.97	2.61–6.45	4.68	< 0.001	0.194	0.185
	Model 2 (Demographics)	4.35	0.97	2.43–6.28	4.49	< 0.001	0.268	0.235
	Model 3 (Full)	3.80	1.06	1.69–5.91	3.58	< 0.001	0.281	0.240
Turn Duration Variability	Model 1 (Unadjusted)	7.49	1.68	4.15–10.83	4.46	< 0.001	0.179	0.170
	Model 2 (Demographics)	7.83	1.74	4.38–11.29	4.50	< 0.001	0.204	0.168
	Model 3 (Full)	5.56	1.83	1.92–9.19	3.04	0.003	0.279	0.238
Turn Velocity Variability	Model 1 (Unadjusted)	3.60	3.19	–2.74–9.94	1.13	0.262	0.014	0.003
	Model 2 (Demographics)	3.81	3.08	–2.31–9.93	1.24	0.219	0.169	0.131
	Model 3 (Full)	0.50	3.29	–6.04–7.03	0.15	0.881	0.222	0.177

SE, standard error; CI, confidence interval; Adj. R², adjusted R-squared. β values are unstandardized regression coefficients; for Models 2 and 3, they represent the partial regression coefficient for T2DM status after adjustment for the covariates included in each model. Model 1: group status only. Model 2: adjusted for age, sex, and BMI. Model 3: additionally adjusted for education and MMSE. All p-values are two-sided.

#### 3.3.1 Model fit and variance explained

Progressive adjustment for confounders improved model fit across all gait variability measures (**Supplementary Figure S4**). Stance variability showed adjusted R² values increasing from 0.258 (Model 1: unadjusted) to 0.264 (Model 2: demographics) to 0.387 (Model 3: full model), indicating that the fully-adjusted model explained 38.7% of the variance. Gait speed variability also demonstrated strong explanatory power, with adjusted R² rising from 0.226 (unadjusted) to 0.304 (full model). Stride length variability and turn duration variability exhibited moderate fits (adjusted R² = 0.240 and 0.238, respectively), whereas turn velocity variability was only weakly predicted (adjusted R² = 0.177).

#### 3.3.2 Effect of T2DM on gait variability

After full adjustment (Model 3), T2DM status was significantly associated with increased variability in all measures except turn velocity (**Supplementary Figure S5**). Individuals with T2DM exhibit consistently higher gait variability independent of age, gender, BMI, education, and baseline cognitive function.

### 3.4 Diagnostic accuracy of gait variability measures

**Figure 2:**
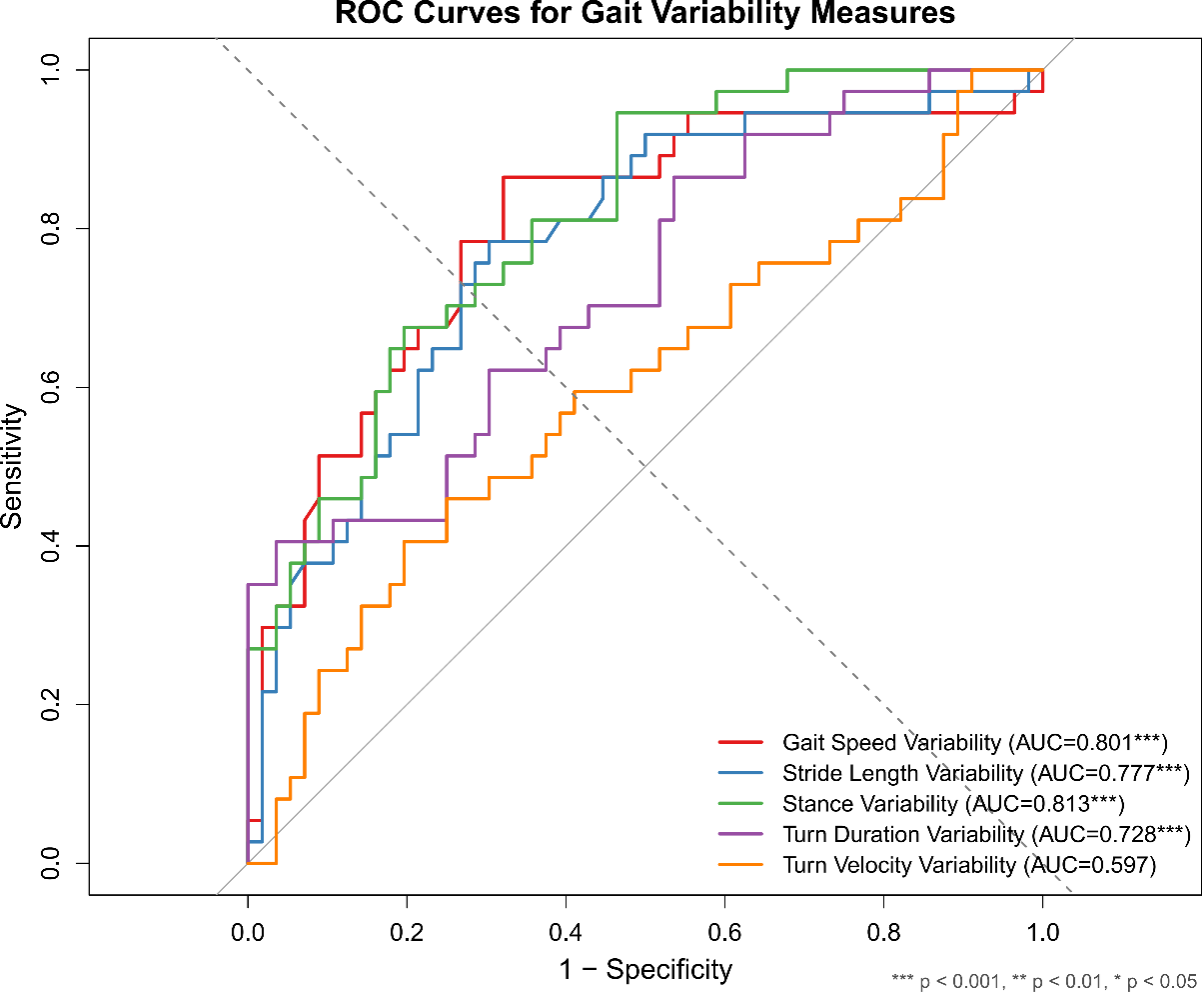
ROC comparisons of gait variability measures show stance and gait-speed variability provide the best discrimination.

ROC curve analysis demonstrated that several gait variability measures had good to fair discriminatory ability for identifying individuals with T2DM (**Figure 2**; **Table 3**).

**Table 3:** Diagnostic performance of gait variability measures for identifying T2DM.

Measure	AUC (95% CI)	Cut-off (%)	Sensitivity (%)	Specificity (%)	*P*-value
Stance Variability	0.813 (0.727–0.898)	2.06	94.6	53.6	< 0.001
Gait Speed Variability	0.801 (0.705–0.897)	10.36	86.5	67.9	< 0.001
Stride Length Variability	0.777 (0.680–0.874)	7.16	78.4	69.6	< 0.001
Turn Duration Variability	0.728 (0.623–0.833)	31.42	40.5	96.4	< 0.001
Turn Velocity Variability	0.597 (0.475–0.719)	19.83	45.9	75.0	0.119

AUC, area under the ROC curve; CI, confidence interval.

Optimal cut-offs determined by Youden’s index.

Stance Variability exhibited the highest diagnostic accuracy (AUC = 0.813, 95% CI: 0.727–0.898, P < 0.001). At the optimal cut-off of 2.06%, sensitivity reached 94.6% and specificity 53.6%. Gait Speed Variability also showed good performance (AUC = 0.801, 95% CI: 0.705–0.897, P < 0.001); the optimal threshold of 10.36% yielded 86.5% sensitivity and 67.9% specificity. Stride Length Variability achieved fair accuracy (AUC = 0.777, 95% CI: 0.680–0.874, P < 0.001) with a cut-off of 7.16% providing 78.4% sensitivity and 69.6% specificity.

Turn Duration Variability demonstrated fair discriminatory ability (AUC = 0.728, 95% CI: 0.623–0.833, P < 0.001), whereas Turn Velocity Variability did not significantly discriminate between groups (AUC = 0.597, 95% CI: 0.475–0.719, P = 0.119).

### 3.5 Mediation analysis

**Figure 3:**
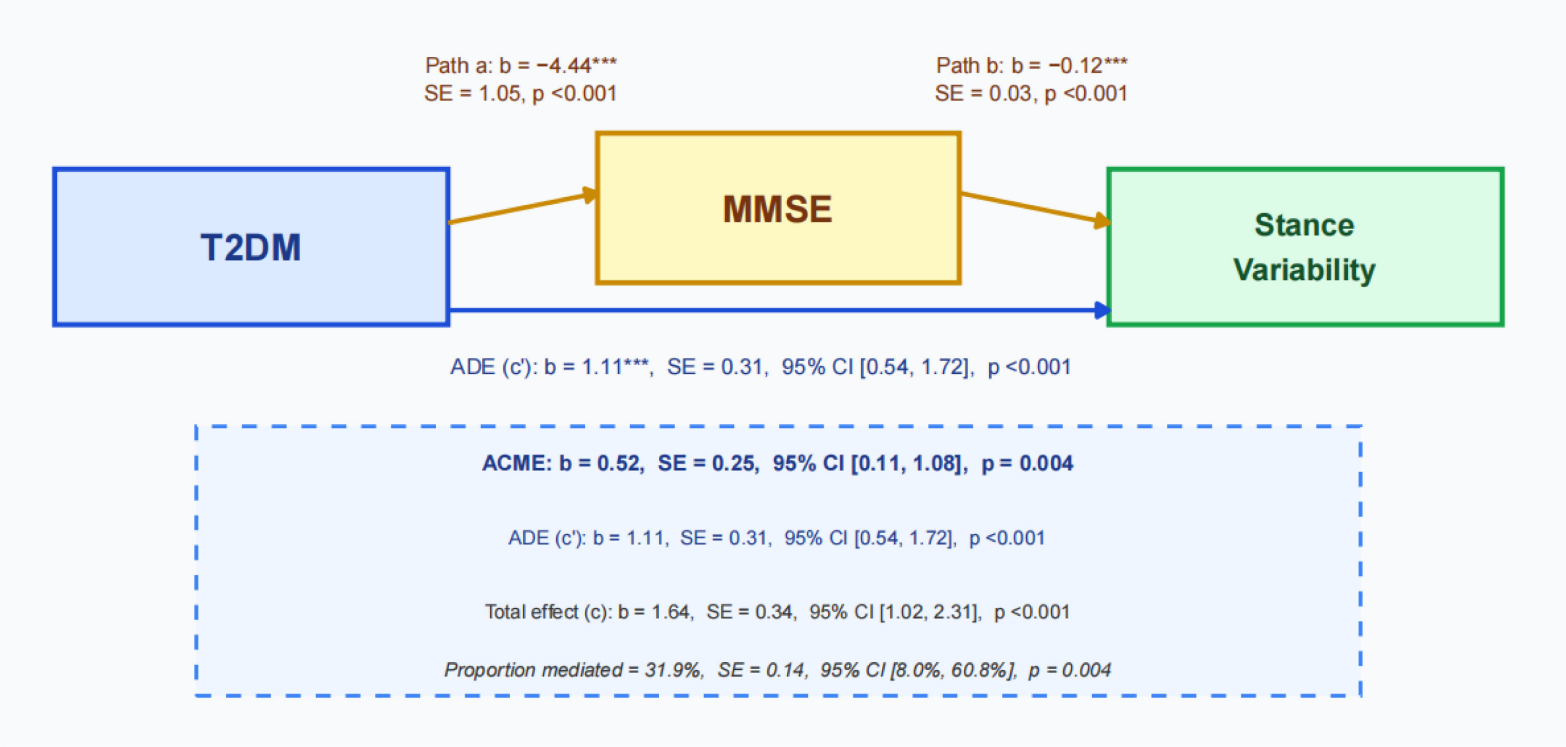
Mediation model of the association between T2DM and stance variability through MMSE. Path a denotes the association between T2DM and MMSE. Path b denotes the association between MMSE and stance variability after adjustment for T2DM. Path c denotes the total effect of T2DM on stance variability estimated without inclusion of MMSE, and path c′ denotes the direct effect after inclusion of MMSE. The indirect effect (ACME), direct effect (ADE), total effect, and proportion mediated were estimated using nonparametric bootstrap procedures with 5,000 resamples. All paths in the primary model were adjusted for age, sex, BMI, and education. ***p < 0.001.

Mediation analyses examined whether cognitive function (MMSE) mediated the relationship between T2DM status and gait variability using a unified regression-based causal mediation framework adjusted for age, sex, BMI, and education (**Figure 3**; **Table 4**). In this framework, the total effect represents the overall association between T2DM and stance variability estimated without inclusion of MMSE, whereas the direct effect represents the association after adjustment for MMSE.

**Table 4:** Mediation analysis of the association between T2DM and stance variability through MMSE.

Effect	Estimate	SE	95% CI	p-value	% mediated
Indirect effect (ACME)	0.523	0.252	0.112–1.083	0.0036	
Direct effect (ADE)	1.114	0.308	0.545–1.725	<0.001	
Total effect	1.637	0.337	1.023–2.313	<0.001	
Proportion mediated	0.319	0.137	0.080–0.608	0.0036	31.9%

ACME, average causal mediation effect; ADE, average direct effect. The total effect represents the overall association between T2DM and stance variability estimated without inclusion of the mediator. The direct effect represents the association between T2DM and stance variability after adjustment for MMSE. The indirect effect represents the mediated effect through MMSE. The primary model was adjusted for age, sex, BMI, and education. Standard errors were estimated as the standard deviation of the nonparametric bootstrap distribution based on 5,000 resamples. A supplementary model without education is provided as a sensitivity analysis.

MMSE partially mediated the effect of T2DM on Stance Variability. The total effect of T2DM on stance variability was β = 1.64 (SE = 0.34, 95% CI: 1.02–2.31, P < 0.001). The indirect effect through MMSE was significant (β = 0.52, SE = 0.25, 95% CI: 0.11–1.08, P = 0.0036), accounting for approximately 31.9% of the total effect. The direct effect remained significant after controlling for MMSE (β = 1.11, SE = 0.31, 95% CI: 0.55–1.73, P < 0.001), indicating partial mediation. In the supplementary analysis without adjustment for education, the indirect effect remained significant, supporting the robustness of the findings. (**Supplementary Table S1**).

## 4 Discussion

The results of this study showed that T2DM older patients exhibited a significant increase in gait variability, including stance time variability (Cohen’s d = 1.22), gait speed variability (Cohen’s d = 1.12), stride length variability (Cohen’s d = 0.99) and turn duration variability (Cohen’s d = 0.94). These findings indicated that gait fluctuations could be more sensitive markers for T2DM-related neuromuscular dysfunction. We found that higher gait variability was consistently correlated with poorer cognitive performance, and the correlation coefficients of key variability indicators ranged from r=−0.20 to r=−0.53 (P<0.05). The stance time variability and gait speed variability demonstrated clinically meaningful diagnostic accuracy, with AUC values of 0.813 and 0.801 respectively. Most notably, the analysis of the mediating effect showed that the cognitive function characterized by MMSE score partially mediated the relationship between the T2DM status and the stance time variability. The indirect effect accounted for 31.9% of the total effect. The significant direct effect still existed, suggesting that T2DM may affect gait variability through multiple pathways, including cognitive decline and non-cognitive mechanisms such as peripheral neuropathy, muscle strength decline, and proprioceptive impairment. Our results support a dual-pathway model, in which T2DM affects gait stability through the central (cognitive) and peripheral (sensorimotor) mechanisms.

Several studies have reported that the gait speed of T2DM patients is reduced, the stride length is shortened, and the double support time is prolonged (26, 27). These changes are considered to be compensatory strategies to maintain stability in the presence of sensory deficits and muscle weakness. Most prior investigations only focused on the average gait parameters and paid limited attention to gait variability. However, gait variability is increasingly recognized as a more sensitive indicator of underlying neurological dysfunction (28, 29). The limited research investigating the gait variability of T2DM remains inconsistent. Some studies reported an increase in the variability of stride time and gait velocity (30), while others found no significant differences (31). Therefore, our findings should be interpreted in the context of this mixed literature rather than as uniformly consistent with all previous studies. These inconsistencies may stem from differences in methodology, including assessment protocols, environmental conditions and measurement techniques. They may also reflect differences in participant characteristics, such as age, diabetes severity, complication burden, and baseline functional status (32, 33). Studies including younger, less impaired, or more heterogeneous diabetic populations may be less likely to detect significant abnormalities in gait variability. By adopting validated wearable inertial sensors, our research can accurately and real-time quantify the gait variability of multiple spatiotemporal domains in straight walking and turning tasks. In this study, the effect sizes we observed (Cohen’s d = 0.94–1.22) were significantly larger than those reported in previous studies (34, 35), which may be related to our focus on the elderly group. In this particular population, age-related physiological changes will synergistically interact with diabetes-related complications, thus amplifying gait dysfunction (36). At the same time, the null findings reported in some earlier studies highlight that diabetes may not affect all gait parameters equally, and that the magnitude of impairment may depend on the instrumentation, and clinical profile of the sample (37). Recent studies have reported largely preserved muscle synergy structure during walking in individuals with T2DM, supporting the notion that complication status may modulate motor control impairment (38). Previous research has predominantly focused on straight-line walking, and our inclusion in the turning task addresses the limitations of the previous gait evaluation paradigm, because turning requires greater postural control, dynamic balance and executive function than straight walking (39, 40). We observed a significant increase in the variation of turning duration in the T2DM group, which supports the view that turning performance could be more sensitive to the risk of neurological disorder and fall (41). In the future, the inclusion of turning assessment into clinical gait assessment may enhance identifying the decline of function earlier in high-risk populations.

The mechanism of increased gait variability of T2DM in older adults may be multifactorial, involving the complex interaction between peripheral sensorimotor impairment and central cognitive dysfunction (42). As one of the most common complications of T2DM, peripheral neuropathy is characterized by progressive degeneration of peripheral nerve fibers, involving sensory and motor pathways. Motor-related neural changes may occur before the abnormality of conventional nerve conduction examination in diabetes (43). T2DM group’s grip strength decreased significantly and the IADL score increased in our study, reflecting the broader impact of diabetes on neuromuscular and physical function. These sensory defects compromise the individual’s ability to recognize and respond to disturbances during walking, thereby leading individuals to rely more on visual and vestibular feedback systems. This instability can be manifested as an increase in gait variability, especially in the stance time, swing time, and other temporal gait parameters. These may reflect the damage to the brain network necessary to maintain gait stability and can be used as a sign of cognitive and cortical dysfunction (44). In addition, microvascular changes associated with diabetes can directly impair muscle function by reducing blood flow and oxygen delivery of skeletal muscle, thus further destabilizing gait performance (45). In this study, we were surprised to find that cognitive function plays a partial mediating role in the diabetes-gait variability relationship, which provides compelling evidence for the “brain mechanism”. T2DM and its metabolic dysregulation are associated with the accelerated brain aging, and neurodegenerative changes will especially disrupt critical circuits closely related to motor control and executive function, including the cerebral cortex, cerebellum and basal ganglia, which are essential for motor control, motor learning and cognitive-motor integration (46, 47). The executive function is essential for adapting the gait to the environmental demands, planning the walking strategy and maintaining the performance of motor tasks, especially when performing cognitive and motor tasks at the same time (48). Impairment in these cognitive domains may reduce the individual’s capacity for real-time gait adjustments, resulting in increased variability (17, 49). The indirect effects observed in our study suggest that cognitive decline plays an important and clinically relevant role in the relationship between diabetes and gait variability. The remaining 71.9% of the effects likely reflect multiple peripheral mechanisms, including diabetic peripheral neuropathy, microvascular dysfunction and decreased muscle strength. Peripheral sensorimotor defects and central cognitive impairment could contribute to gait instability independently and synergistically. Importantly, the proportion of cognitive function mediated was relatively large (31.9%), which underscores the critical significance of cognitive-motor integration in maintaining gait stability. This result suggests that cognitive interventions may benefit for fall prevention in the older populations with T2DM. Based on existing research, we have reason to believe that interventions targeting either pathway (such as improving peripheral deficits through glycemic control and neuropathy management, or cognitive training and physical exercise to improve executive function) may offer a complementary strategy to help preserve gait stability and reduce the fall risk (50, 51).

In this study, our results indicate that gait variability parameters, especially stance time variability and gait speed variability, have high diagnostic accuracy (AUC = 0.813 and 0.801), suggesting that these indicators can be used to identify individuals with decreased motor function and high risk of falling. The optimal cut-off value of stance time variability is 2.06%, and its sensitivity is 94.6%, indicating that this metric can effectively capture most high-risk individuals. The gait variability evaluation based on wearable inertial sensors has many advantages over traditional clinical measurements. It can provide continuous and real-time data under natural walking conditions; it can be implemented in a variety of scenarios such as clinics, community centers and home; less training is required, which is scalable for wide promotion (52, 53). We observed strong correlations between gait variability and cognitive function, indicating that gait assessment may also serve as a functional biomarker for T2DM cognitive decline. Cognitive screening in routine T2DM care is still underutilized. It is therefore recommended to incorporate yearly cognitive screening using validated tools such as MMSE or MoCA into the routine management of ≥65-year-old T2DM patients, because cognitive dysfunction will significantly impair the diabetes self-management and increase the risk of adverse outcomes (54). Existing evidence shows that the dual decline of walking speed and cognition has the strongest association with the risk of dementia, thus supporting the inclusion of gait speed in dementia risk screening. This approach is also consistent with emerging concepts of “motor cognitive risk syndrome”, which emphasizes that the simultaneous decline of motor function and cognition is a pre-dementia syndrome that requires clinical attention (55, 56). Adding a brief gait assessment to the T2DM outpatient follow-up can identify patients who may require further formal cognitive evaluation and targeted intervention at an early stage. Based on our identification of cognitive function as a partial mediator of the diabetes–gait variability relationship, treatments targeting peripheral deficits (optimized glycemic control, neuropathy management and resistance training) and central defects (dual-task multimodal training, combination of multisensory cognitive stimulation and moderate-intensity physical exercise) may be most conducive to maintaining mobility and preventing falls (57, 58). In addition, we observed a significant increase in the turn duration variability in the T2DM group, highlighting the importance of incorporating turning evaluation into clinical gait assessments. Fallers in prospective follow-up often show reduced turning frequency, prolonged turn duration, and greater variability of the consistency of turn angle (59). Simple turning tests, such as the Timed Up and Go tests and the 360-degree turn test, can be used as part of routine fall risk screening. Our findings support a shift toward a more comprehensive and technologically enhanced gait assessment paradigm in T2DM, which is expected to improve the early identification of high-risk individuals and guide personalized intervention strategies.

This study has several limitations that need to be considered. The cross-sectional design precludes causal inference. Considering the differences between groups in educational attainment, we used the MMSE (not the MoCA) as the cognitive measure in the mediation analysis to reduce education-related bias. Longitudinal studies are needed to establish the temporal relationship between T2DM, cognitive function and gait variability. The gait assessment based on hospital may not fully reflect the complexity of walking in the real world. Our samples come from a single region and do not contain serious complications, which may limit the generalizability. Participants were relatively high-functioning older outpatients who could walk independently for 10 minutes. Thus, our findings may be more applicable to ambulatory older adults with T2DM than to frailer or more impaired patients. Excluding individuals with severe dysfunction may introduce selection bias. Accordingly, the gait variability observed here may underestimate or differ from that in diabetic populations with greater frailty or mobility impairment. We also did not systematically assess the severity of peripheral neuropathy or the impact of psychological factors (such as depression and anxiety) on gait variability. The inclusion of these variables in future research will help to establish a more comprehensive model of T2DM gait instability and multiple determinants. Despite the above limitations, this study still provides relatively solid evidence for the elevation of gait variability in the older adults with T2DM, and clarifies some of the mediating role of cognitive function, which provides important insights for mechanistic understanding and clinical application.

In summary, this study demonstrates that the gait variability of the older adults with T2DM has increased significantly. Cognitive function partially mediated this relationship (31.9%), supporting the dual-pathway model involving peripheral sensorimotor and central cognitive deficits. The stance time variability and gait speed variability showed high diagnostic accuracy (AUC>0.8), suggesting potential utility as screening markers. Our results support that multi-component interventions for metabolic control and cognitive-motor function may be the most effective for maintaining the mobility of this population and reducing the risk of falling. Future longitudinal research that combines comprehensive neuropsychological assessment, quantitative neuropathy measures and gait monitoring in complex real-world environments are needed. These studies should help establish a causal relationship, clarify the mechanism, and verify the clinical utility of gait variability in predicting falls and multidimensional function decline in the older adults with T2DM.

## Data Availability

The raw data supporting the conclusions of this article will be made available by the authors, without undue reservation.
