# Supplementary material for: Gait variability as a dual-pathway marker of cognitive-motor dysfunction in older adults with type 2 diabetes mellitus
Source: Front Endocrinol (Lausanne). 2026 Mar 31;17:1814718. doi: 10.3389/fendo.2026.1814718 (PMC13076179; doi:10.3389/fendo.2026.1814718)
Supplement: Supplementary file 1 [file DataSheet1.docx]

**Table S1.Supplementary model**

| **Effect** | **Estimate** | **SE** | **95% CI** | **p-value** | **% Mediated** |
| --- | --- | --- | --- | --- | --- |
| Indirect effect (ACME) | 0.449 | 0.246 | 0.043–0.998 | 0.028 |  |
| Direct effect (ADE) | 1.148 | 0.312 | 0.551–1.771 | <0.001 |  |
| Total effect | 1.597 | 0.327 | 0.976–2.262 | <0.001 |  |
| Proportion mediated | 0.281 | 0.142 | 0.030–0.589 | 0.028 | 28.1% |

**Figure S1. Between-group differences in gait variability measures**


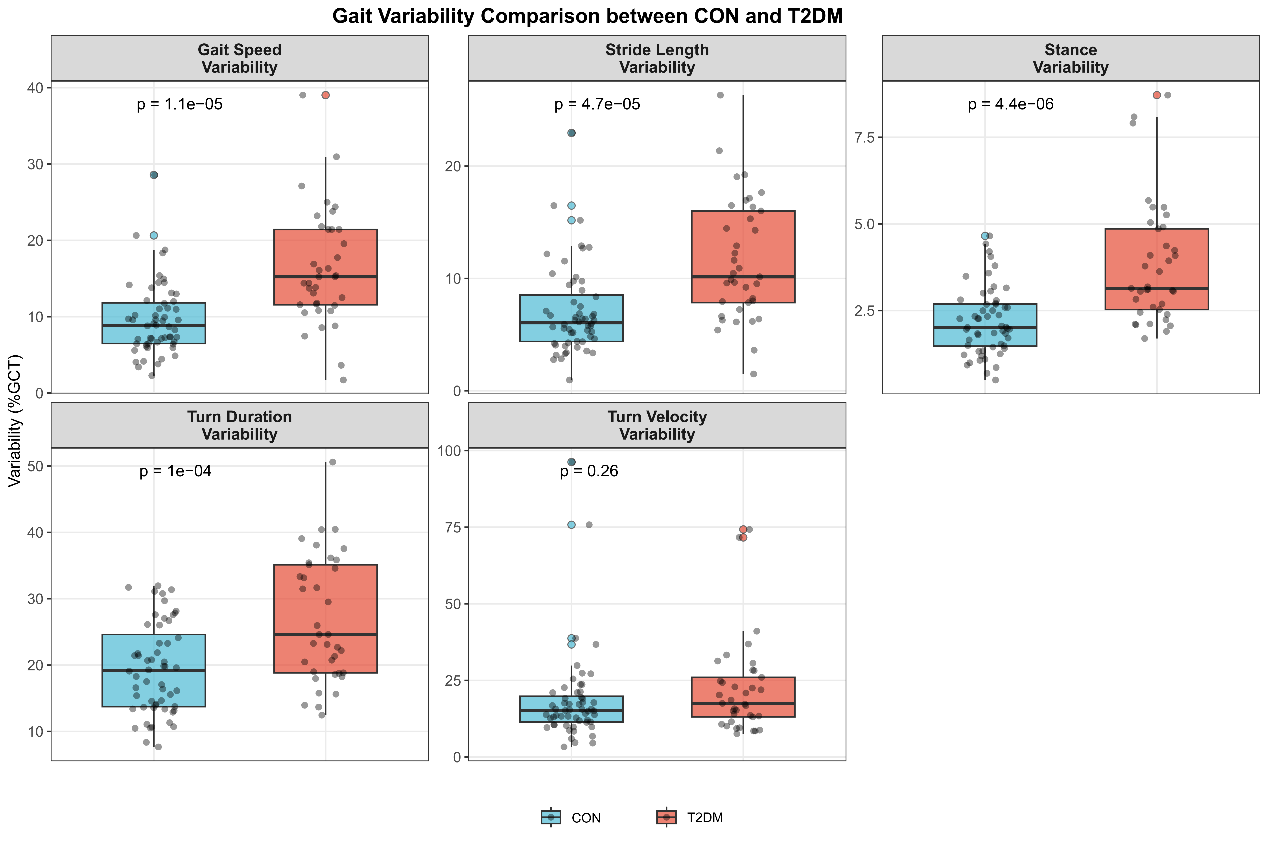


**Figure S2. The effect sizes (Cohen’s d) for each gait variability**


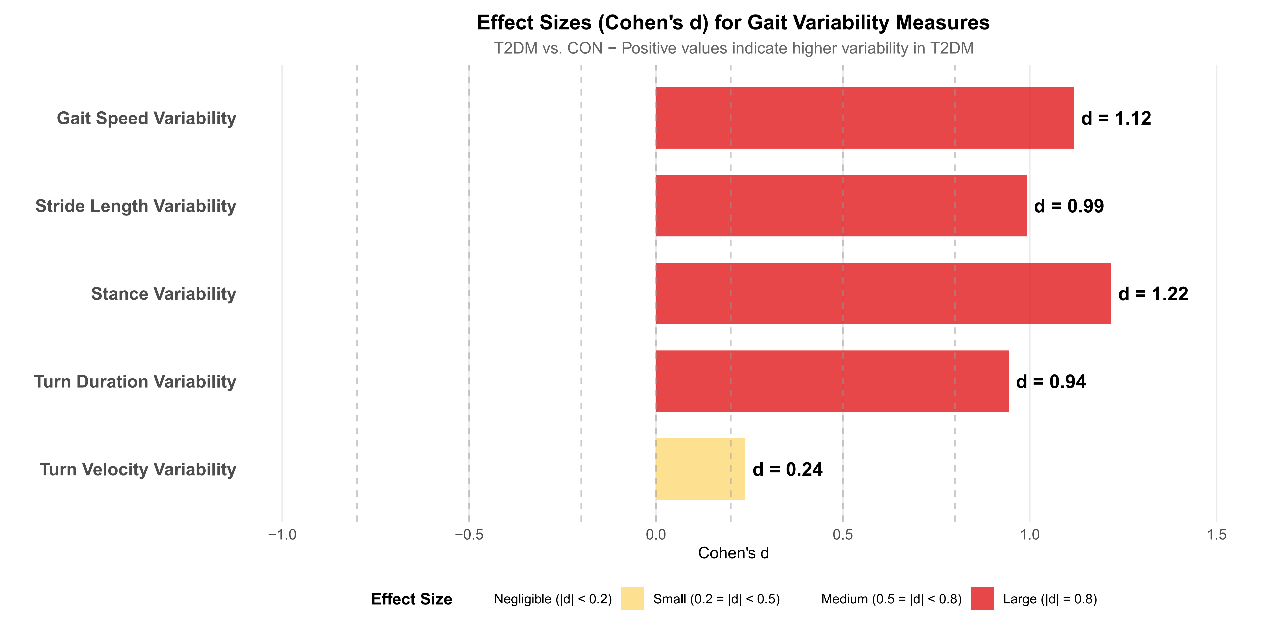


**Figure S3. Correlation Heatmap between Gait Variability and Cognitive Function**


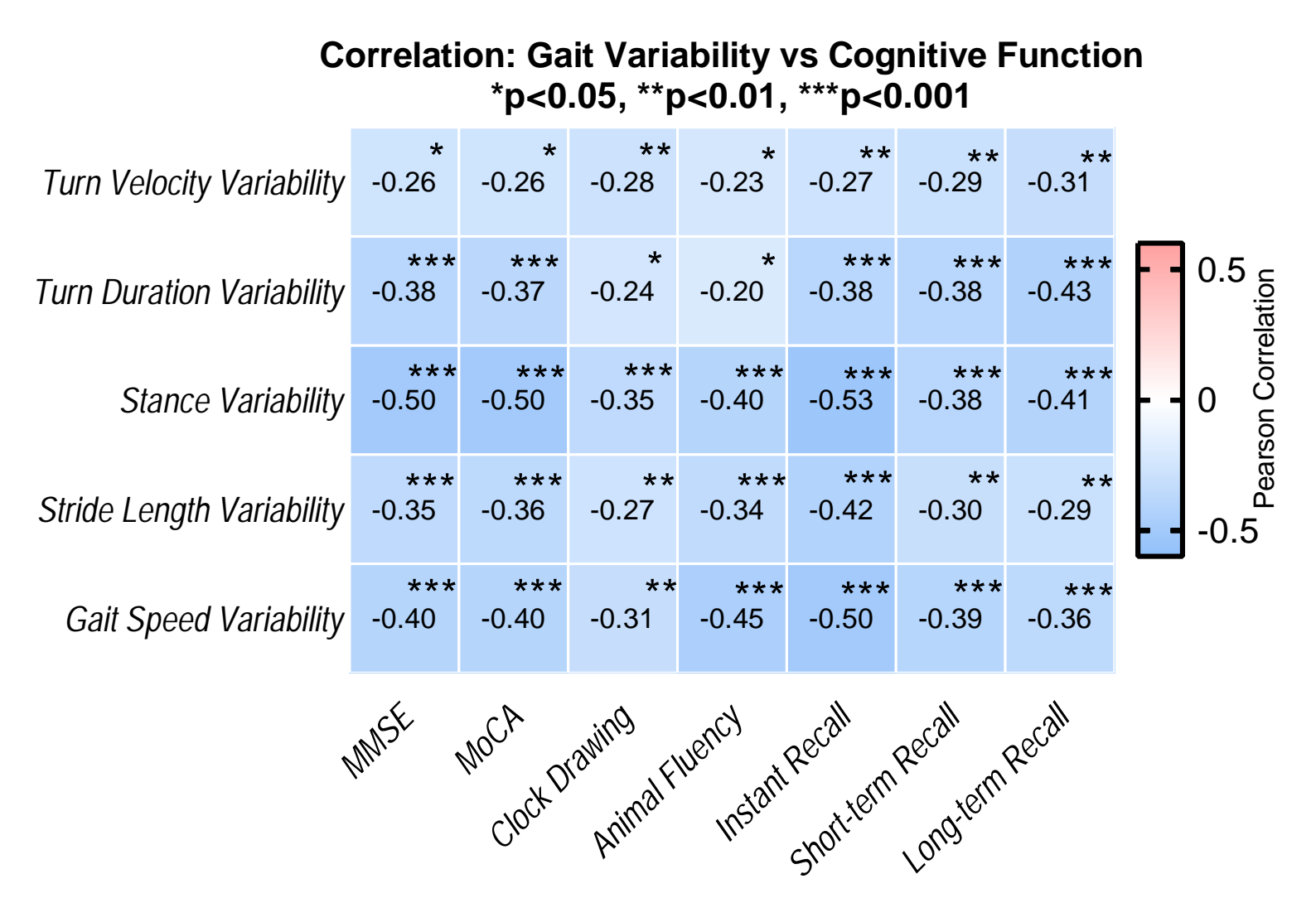


**Figure S4. Improvement in model fit with progressive covariate adjustment.**


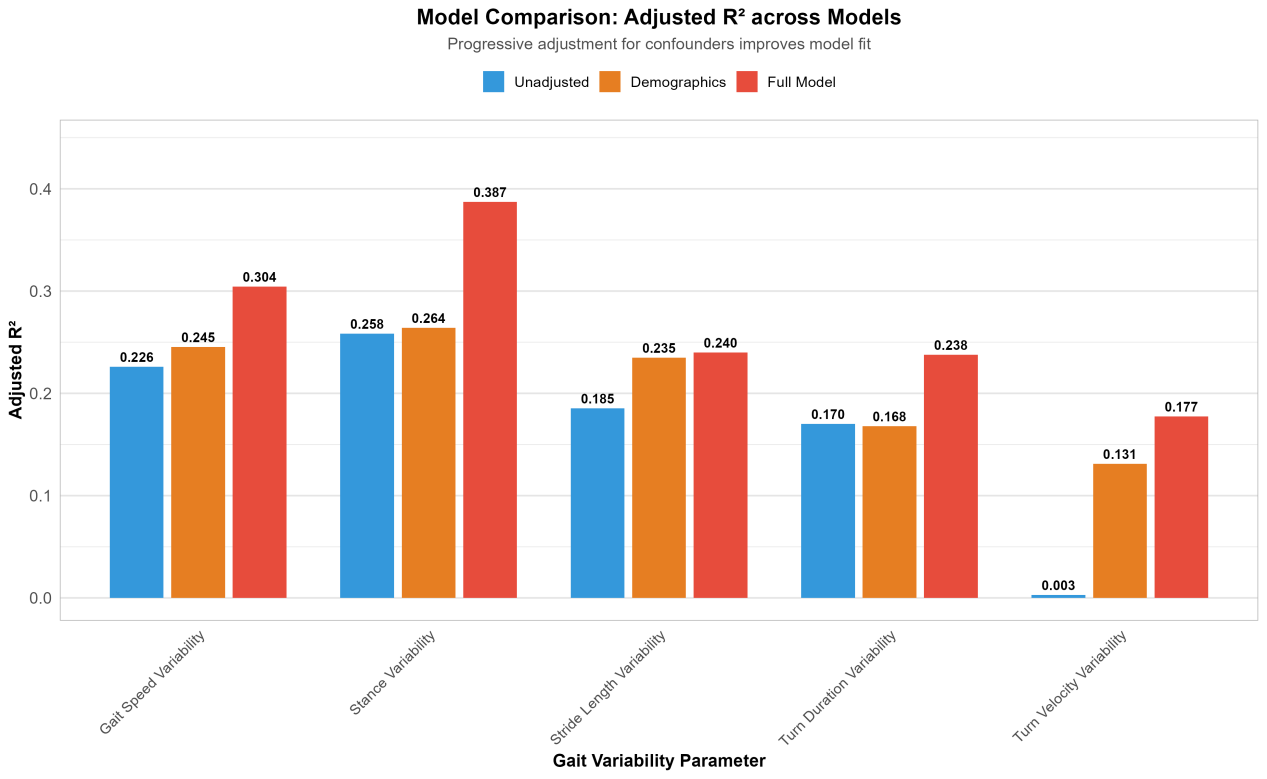


**Figure S4.** Adjusted R² values across the three hierarchical regression models (unadjusted, demographics, full model) for each gait variability outcome.

**Figure S5. Adjusted effect of T2DM on gait variability measures (Model 3).**


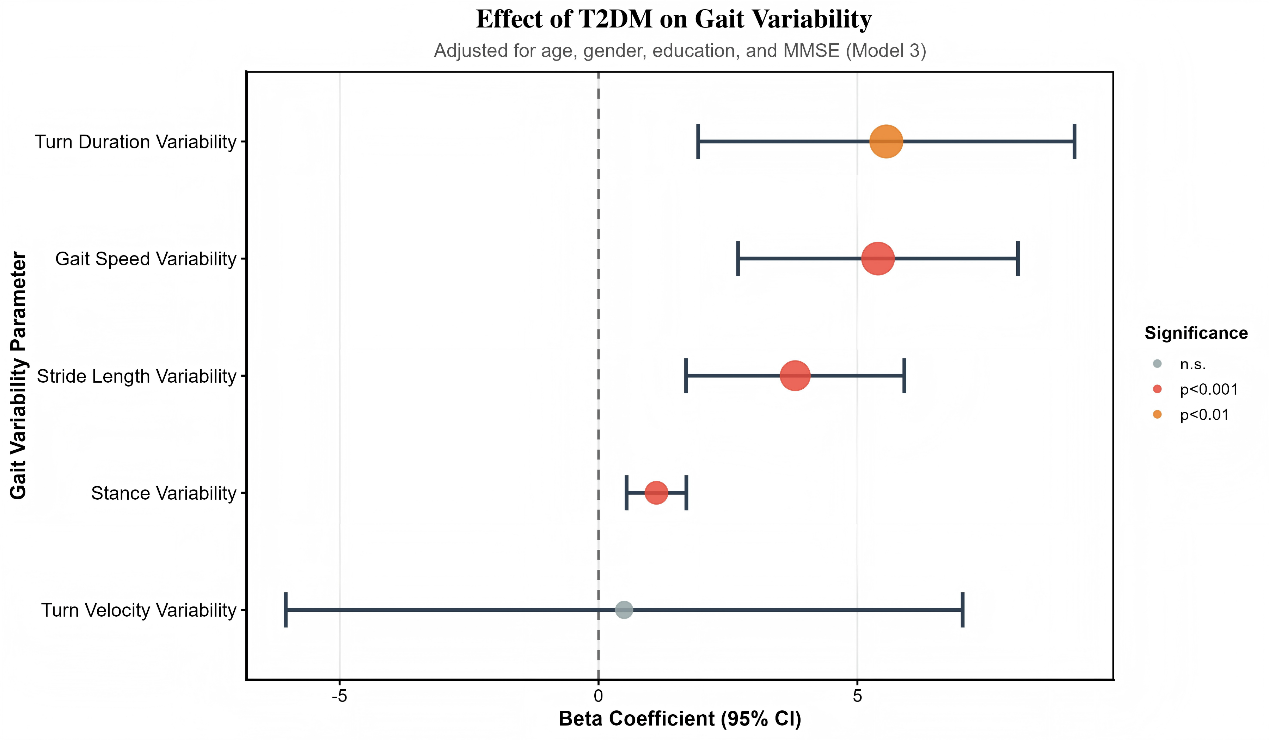


**Figure S5.** Forest plot shows regression coefficients (β) and 95 % CI after adjustment for age, gender, BMI, education and MMSE; significance levels are indicated by P < 0.01 and P < 0.001.
